# Frontiers in Plasma Proteome Profiling Platforms: Innovations and Applications

**DOI:** 10.21203/rs.3.rs-4193960/v1

**Published:** 2024-04-01

**Authors:** Rajesh Kumar Soni

**Affiliations:** Columbia University Irving Medical Center

**Keywords:** Biomarker, Biofluids, Secretome, Plasma proteome, Seer Proteograph, SP100 Instrument, PreOmics-iST, timstofPro2

## Abstract

Biomarkers play a crucial role in advancing precision medicine by enabling more targeted and individualized approaches to diagnosis and treatment. Various biofluids, including serum, plasma, cerebrospinal fluid (CSF), saliva, tears, pancreatic cyst fluids, and urine, have been identified as rich sources of potential for the early detection of disease biomarkers in conditions such as cancer, cardiovascular diseases, and neurodegenerative disorders. The analysis of plasma and serum in proteomics research encounters challenges due to their high complexity and the wide dynamic range of protein abundance. These factors impede the sensitivity, coverage, and precision of protein detection when employing mass spectrometry, a widely utilized technology in discovery proteomics. Conventional approaches such as neat plasma workflow are inefficient in accurately quantifying low-abundant proteins, including those associated with tissue leakage, immune response molecules, interleukins, cytokines, and interferons. Moreover, the manual nature of the workflow poses a significant hurdle in conducting large cohort studies. In this study, our focus is on comparing workflows for plasma proteomic profiling to establish a methodology that is not only sensitive and reproducible but also applicable for large cohort studies in biomarker discovery. Our investigation revealed that the SeerProteographXT workflow outperforms other workflows in terms of plasma proteome depth, quantitative accuracy, and reproducibility while offering complete automation of sample preparation. Notably, SeerProteographXT demonstrates versatility by applying it to various types of biofluids. Additionally, the proteins quantified widely cover secretory proteins in peripheral blood, and the pathway analysis enriched with relevant components such as interleukins, tissue necrosis factors, chemokines, and B and T cell receptors provides valuable insights. These proteins, often challenging to quantify in complex biological samples, hold potential as early detection markers for various diseases, thereby contributing to the improvement of patient care quality.

## Background

Biomarkers, encompassing measurable substances, structures, or biological processes in the body, play a crucial role in various aspects of medical research and clinical applications. They are integral to disease diagnosis, prognosis, and monitoring, as well as drug development and the emerging field of personalized medicine^1,2^. The identification and validation of biomarkers involves advanced technologies, including genomics, proteomics, metabolomics, and imaging techniques. Tissue biopsy and diverse biofluids, such as serum, plasma, cerebrospinal fluid (CSF), saliva, tears, pancreatic cyst fluids, and urine, are considered rich sources of biomarkers^3^.

The burden of cardiovascular diseases (CVDs) stands as the leading cause of death in the United States. In the United States alone, CVD accounted for 695,000 deaths in 2021, representing 1 in every 5 deaths^4,5^. Concurrently, a recent United Nations report indicates that nearly one billion people, or 1 in every six individuals worldwide, suffer from neurological disorders, encompassing conditions such as Alzheimer’s and Parkinson’s diseases, strokes, multiple sclerosis, epilepsy, migraines, brain injuries, and neuro-infections, contributing to approximately 6.8 million deaths annually^6^.

The global health challenges extend to cancer, which ranks as the second leading cause of death worldwide and is responsible for 1 in 6 deaths worldwide^7^. Early detection is crucial for reducing the cancer burden, with the potential to lower cases by 30 to 50% through risk factor avoidance, evidence-based prevention strategies, and early detection methods^7^. Biomarkers contribute to early detection, prognosis, and personalized treatment strategies for CVD, neurological disorders, and cancer. CVD biomarkers such as cardiac troponin (cTn), High-sensitivity cardiac troponin (hs-cTn), High-sensitivity Creactive protein (hs-CRP) in peripheral blood quanti es markers for myocardial injury, and natriuretic peptides (BNP or NT-proBNP) for heart failure^8^.

Currently, there is no clinical test that can de nitively diagnose ALS or FTD; a recent study detected abnormal proteins, specifically TDP-43 dysfunction in the spinal fluid of individuals with amyotrophic lateral sclerosis (ALS) and frontotemporal dementia (FTD), potentially serving as a protein biomarker to improve diagnosis^9^.

Understanding and harnessing the potential of protein biomarkers across these major health concerns can significantly impact disease management and patient outcomes on a global scale. Therefore, the exploration of innovative protein biomarker discovery workflows, as outlined in the subsequent discussion, holds promise for advancing the understanding and management of these widespread and impactful health conditions.

Traditionally, tissue biopsy has been a cornerstone for cancer diagnosis, offering histological and mutational pro les. However, this method is invasive and presents challenges related to sample accessibility, repetition frequency, patient comorbidities, tissue storage, and sample integrity maintenance^3^. Overcoming these barriers, various biofluids collected non-invasively, such as blood, urine, saliva, or cerebrospinal fluid, offer potential alternatives. Nevertheless, the high complexity and large dynamic range of protein abundance in these fluids pose challenges to mass spectrometry analysis, hindering sensitivity, coverage, and precision^10^.

To address these challenges, this study evaluates three distinct workflows—NeatPlasma, PreOmicsEnrich-iST, and SeerProteographXT—for their applicability in biomarker discovery. The assessment focuses on reproducibility, robustness, and the ability to achieve comprehensive proteome coverage, utilizing human pool plasma samples as a representative model. Among these workflows, the SeerProteographXT outperforms others, while also offering complete automation of sample preparation, thereby providing a promising avenue for advancing early detection disease biomarker discovery.

## Methods

### Neat Plasma sample preparation

1 μL of neat plasma samples was diluted at a 1:10 ratio with 100 mM TrisHCl, pH 8.5. Subsequently, 1.5 μL of the diluted plasma samples were resuspended in 40 μL of freshly prepared SDC lysis buffer^11^ (1% SDC and 100 mM TrisHCl, pH 8.5) and boiled for 15 minutes at 60°C, 1200 rpm for denaturation. Protein reduction and alkylation of cysteines were carried out using 10 mM TCEP and 40 mM CAA for 10 minutes at 45°C, 1200 rpm followed by sonication in a water bath, cooled down to room temperature. Protein digestion was performed overnight by adding LysC/trypsin mix in a 1:50 ratio (μg of enzyme to μg of protein) at 37°C and 1400 rpm. The resulting peptides were acidi ed by adding 1% TFA, vortexed, and subjected to StageTip clean-up via SDB-RPS^11^, followed by drying in a speed-vac. The peptides were then resuspended in 10 μL of LC buffer (3% ACN/0.1% FA). Peptide concentrations were determined using NanoDrop, and 200 ng of each sample was utilized for diaPASEF analysis on timsTOFPro2.

### Plasma sample preparation with PreOmicsEnrich-iST

Plasma samples were processed using the PreOmics ENRICH-iST Kit following the vendor’s provided protocols^12^. In brief, 20 μL of plasma samples were incubated with pre-washed EN-BEADS for 30 minutes at 30°C and 1,200 rpm in 1.5 mL Eppendorf tubes on a ThermoMixer with EN-BIND buffer. Proteins bound to EN-BEADS were washed three times, and the proteins were further processed using the iST-BCT workflow, optimized for biofluids. Next, 50 μL of LYSE-BCT was added to each Eppendorf tube, and the samples were heated at 95°C for 10 min with agitation at 1,200 rpm. After cooling the Eppendorf tubes to room temperature, trypsin digestion buffer was added, and the tubes were incubated at 37°C for 3 h with shaking at 1200 rpm. The digestion process was stopped by adding the supplied stop buffer, and the remaining reaction supernatant was cleaned up using the provided lter cartridge. The peptides were eluted twice with 100 μL of elution buffer and combined. The peptide concentration was measured using NanoDrop, and 200 ng of each sample was utilized for diaPASEF analysis on timsTOFPro2.

### Plasma sample preparation with Seer’s Proteograph Assay

240 μL plasma samples were used. The corona formation, wash, protein lysis and alkylation, digestion, and peptide cleanup were done on SeerProteographXT workflow on SP100 Automation Instrument (Seer) as described^13^ After peptide elution, peptide concentration was measured by a quantitative uorometric peptide assay kit from Thermo Fisher Scientific (Waltham, MA, USA). The peptides were then dried using a Speed Vac. Finally, the dried peptides were reconstituted in the provided reconstitution buffer to a concentration of 200 μg/μL. 200 ng of each sample was utilized for diaPASEF analysis on timsTOFPro2.

### Liquid chromatography with tandem mass spectrometry (LC-MS/MS)

Peptides were separated within 87 min at a ow rate of 400 nl/min on a reversed-phase C18 column with an integrated CaptiveSpray Emitter (25 cm × 75μm, 1.6 μm, IonOpticks). Mobile phases A and B were with 0.1% formic acid in water and 0.1% formic acid in ACN. The fraction of B was linearly increased from 2 to 23% within 70 min, followed by an increase to 35% within 10 min and a further increase to 80% before re-equilibration. The timsTOF Pro2 was operated in diaPASEF mode^14^ and data was acquired at defined 32 × 50 Th isolation windows from m/z 400 to 1,200. To adapt the MS1 cycle time in diaPASEF, set the repetitions to 2 in the 16-scan diaPASEF scheme. The collision energy was ramped linearly as a function of the mobility from 59 eV at 1/K0 = 1.6 Vs cm^− 2^ to 20 eV at 1/K0 = 0.6 Vs cm^− 2^.

### Data analysis

The acquired diaPASEF raw les were searched using the UniProt Human proteome in the DIA-NN 1.8.2 beta^15^ search engine, employing the default settings of the library-free search algorithm with match-between-runs (MBR) enabled. The false discovery rate (FDR) was set to 1% at both the peptide precursor and protein levels.

Results obtained from DIA-NN underwent further statistical analyses and data visualizations using R software version 4.2.3 and RStudio version 2023.12.0 + 369. The R software packages utilized were clusterPro ler, protti, ggplot2, tidyverse, RColorBrewer, and patchwork.

## Results

### A Comprehensive Comparison of Workflows for Plasma Proteome Pro ling

**Figure 1:**
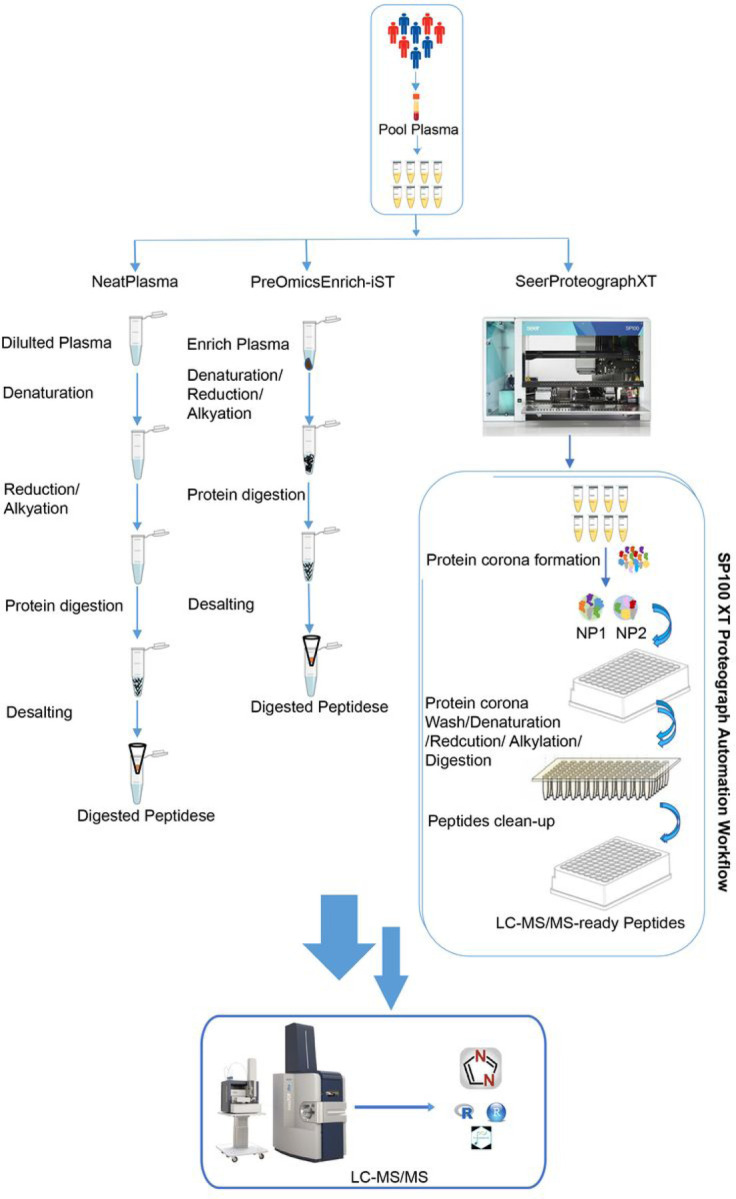
Plasma sample preparation workflows. A visual representation comparing the NeatPlasma, PreOmicEnrich-iST, and SeerProteographXT workflows step by step.

Early disease detection relies on the identification and quantification of reliable biomarkers. The pooled human plasma samples were divided into 8 aliquots, and each aliquot underwent processing to evaluate the NeatPlasma workflow and commercially available sample preparation kit PreOmicsEnrich-iST and the fully automated SeerProteographXT workflow, as depicted in (Fig. 1). The NeatPlasma workflow entails manual processing with laboratory reagents, while the PreOmicsEnrich-iST approach enriches proteins, providing a streamlined sample preparation workflow. In contrast, the SeerProteographXT workflow is fully automated and utilizes two nanoparticles, selectively enriching an unbiased subset of proteins in complex plasma samples.

All three workflows (Fig. 1) were processed using identical pooled plasma aliquots, and data acquisition was conducted on a timsTofPro2 instrument with a 60-minute gradient and diaPASEF method. The subsequent data analysis was performed employing DIA-NN. Initially, the protein identification performance of each workflow was assessed. Across all three workflows, approximately 5881 protein groups were identified. Notably, SeerProteographXT exhibited superior performance, identifying, and quantifying over 4.2-fold more protein groups compared to NeatPlasma and 2.4-fold more compared to PreOmicsEnrich-iST (Supplementary Material 1, Fig. 2A). Similarly, 66987 peptides were identified, with SeerProteographXT quantifying over 6.7-fold more compared to NeatPlasma and 4-fold more compared to PreOmicsEnrich-iST (Supplementary Material 2, Fig. 2B).

The protein dynamic range and complexity play crucial roles in the depth of the quantified plasma proteome, with NeatPlasma samples providing the least information. However, PreOmicsEnrich-iST exhibits improvement compared to NeatPlasma, and the SeerProteograph workflow outperforms both alternatives.

**Figure 2:**
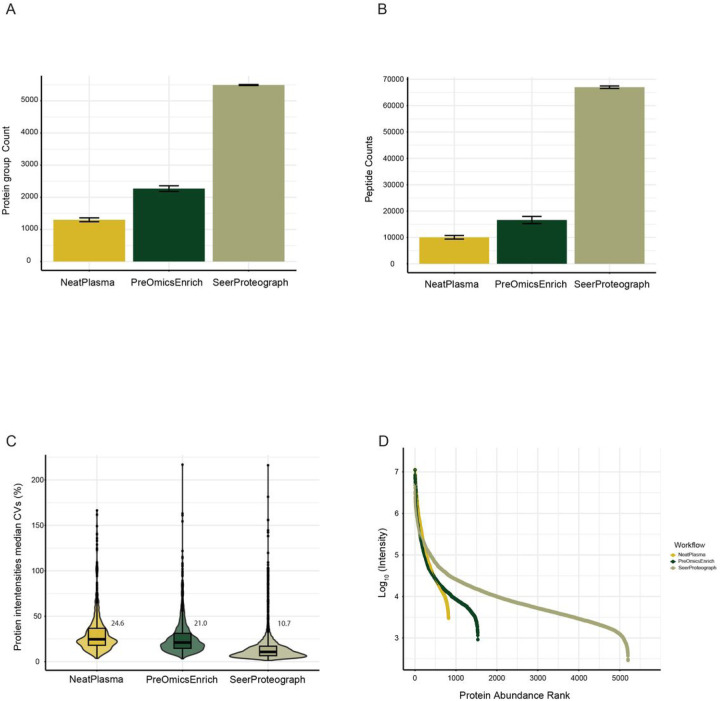
Plasma sample preparation workflow comparison. (A) Protein groups identified by each workflow (B) Peptides identified by each workflow. (C) Coefficient of Variation (CV) for median quantified protein intensities within workflows. (D) The dynamic range of quantified protein abundance across workflows.

Large cohort studies rely on a robust and reproducible workflow. We compared the quantified normalized intensity of protein groups within different workflows NeatPlasma, PreOmicsEnrich-iST, and SeerProteographXT. The NeatPlasma, PreOmicsEnrich-ST, and SeerProteographXT workflows yielded a median coefficient of variation (CV) of 24.6, 21.0, and 10.7%, respectively, as shown in (Fig. 2C). The SeerProteograph workflow demonstrated the lowest CV compared to the NeatPlasma and PreOmicsEnrich-iST workflows, attributed to the uniform and consistent enrichment of proteins using SeerProteograph’s nanoparticle technology, operating across a large dynamic range. The fully automated capabilities of SeerProteograph also contribute to minimizing technical challenges in the workflow.

Plasma/serum samples are complex due to the broad dynamic range of proteins, posing challenges for the identification and quantification of low-abundant proteins through LC-MS/MS. To assess the dynamic range covered by each workflow, we utilized a protein abundance ranking of protein groups’ normalized intensities, revealing an approximate span of 4.6 orders of magnitude. The SeerProteographXT workflow significantly increased the number of quantified proteins by over 6.3-fold and 3.4-fold compared to the NeatPlasma and PreOmicsEnrich-iST workflows. This extension indicates a highly efficient reduction of the dynamic range (Fig. 2D) compared to the NeatPlasma and PreOmicsEnrich-iST workflows.

### Comparative Analysis of Workflows for Secretome Database Coverage

Next, we explored the coverage of the secretome database, which comprises soluble proteins and secreted extracellular vesicles, encompassing biologically active factors such as cytokines, interleukins, interferons, chemokines, complement and coagulation factors, hormones, growth factors, enzymes^16^. These proteins, shed from cells/tumors, play a crucial role in cell signaling, communication, and growth, and their abundance changes under various pathological conditions. While these proteins are secreted into the extracellular space, they are generally more abundant in biological fluids^17,18^. The dynamic nature of secretome protein composition makes them a valuable source of potential biomarkers for cancer and other diseases, aiding in diagnosis, prognosis, and therapeutic monitoring^18^.

**Figure 3:**
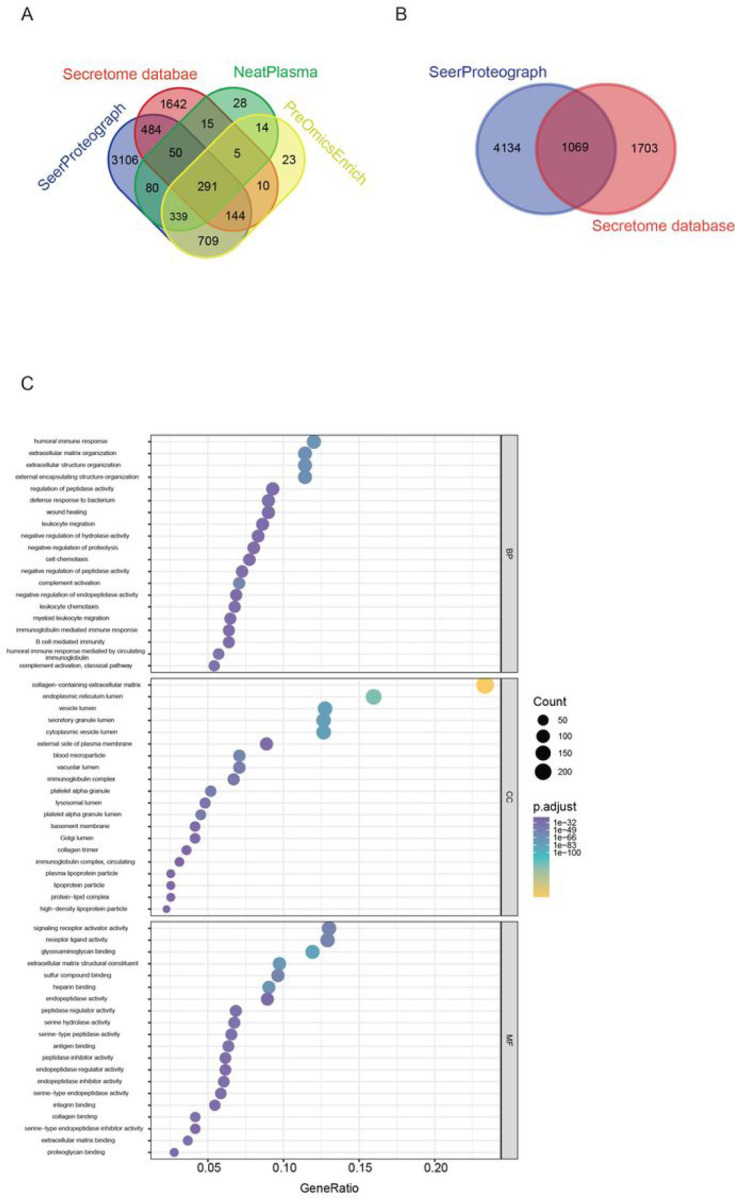
Secretome Protein Database Coverage. (A) Evaluation of protein coverage from the Secretome protein database across workflows. (B Assessment of the percentage overlap between SeerProteograph and Secretome database protein groups. (C) Gene Ontology (GO) enrichment analysis for protein groups overlapping between SeerProteograph and Secretome database.

The Secretome database, sourced from The Human Protein Atlas^19^, underwent a comprehensive comparison across the NeatPlasma, PreOmicsEnrich-iST, and SeerProteographXT workflows to assess coverage. Proteins quantified in all samples within these workflows were included in the analysis, revealing that the SeerProteographXT workflow exhibited notably high coverage, particularly in the quantification of low-abundant proteins (Figure. 3A).

For Gene Ontology (GO) terms functional analysis, a ~ 39% overlap of proteins of SeerProteographXT workflow was chosen (Figure. 3B). This analysis encompassed Molecular Function (MF), Biological Processes (BP), and Cellular Compartments (CC) (Figure. 3C). The proteins predicted to be secreted into human blood encompassed a diverse array, including well-characterized proteins associated with the extracellular matrix organization, enzymes, receptors, cytokines, complement activation, peptidase activator, humoral immune response, wound healing, leukocyte migration, cell chemotaxis, myeloid leukocyte migration, transport proteins, developmental proteins, defense proteins, enzymes, enzyme inhibitors, integrin binding, antigen binding, glycosaminoglycan binding, collagen binding, B cell-mediated immunity-related proteins, and classical pathway.

These identified proteins were found in various cellular compartments, including the endoplasmic reticulum (ER) lumen, vesicle lumen, secretory granule lumen, blood microparticles, lysosomal lumen, platelet alpha granule lumen, Golgi lumen, plasma lipoprotein particles, and protein-lipid complexes.

### Comparative Analysis of Workflows for Functional Annotation Coverage

**Figure 4:**
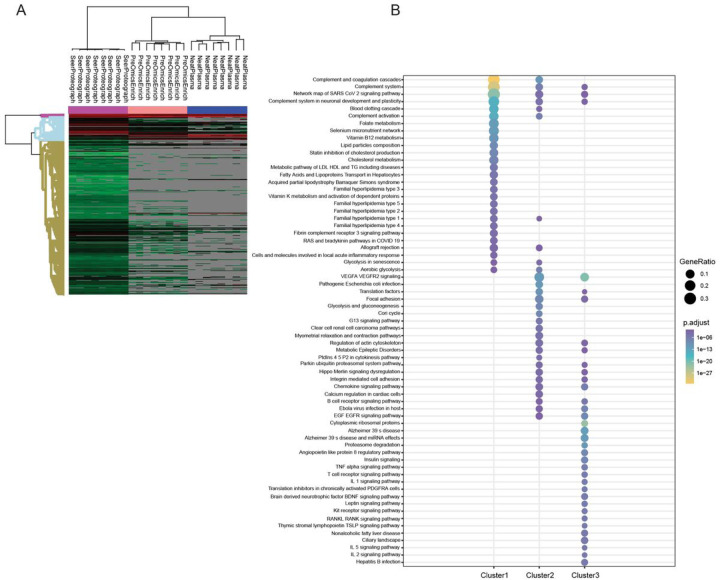
Functional Annotation Coverage. (A) Hierarchical clustering of normalized Log2 protein intensities (B) Pathway analysis of enriched proteins in each identified cluster using the ClusterProfiler package.

We investigated the coverage of proteins quantified in three workflows using functional annotation enrichment analysis. Hierarchical clustering of quantified proteins based on their log2 intensity yielded three distinct groups of clusters (Figure.4A). Each cluster was analyzed for enriched pathways using ClusterProfiler R package of the function of compareCluster with WikiPathways^20^ using a threshold of Benjamini and Hochberg (BH) adjusted p-value < 0.05. Proteins covered with cluster 1 showed significant enrichment for a variety of pathways including complement and coagulation cascades, complement system, complement activation, blood clotting cascade, lipid particle composition, cholesterol metabolism, metabolism of triglycerides, and acute inflammatory response. Proteins present in Cluster 1, quantified in all three workflows, these proteins are highly abundant and consistently quantified.

Proteins associated with EGF EGFR signaling, VEGFA VEGFR2 signaling, glycolysis and gluconeogenesis, chemokine signaling pathway, and B cell receptor signaling pathway are enriched by cluster 2. Proteins present in Cluster 2, quantified in PreOmicsEnrich-iST, and SeerProteographXT workflows.

Proteins associated with Insulin signaling, TNF alpha signaling pathway, T and B cell receptor signaling, IL1/2/5 signaling, proteasome degradation pathways were enriched by cluster 3. Cluster 3 proteins were identified in SeerProteographXT workflow only, these proteins are low abundant in the samples and could potentially serve as crucial biomarkers.

## Discussion

The emergence of cutting-edge technologies for discovery-based quantitative proteomics, such as ultra-sensitive and high-speed mass spectrometers, fully automated sample preparation systems, and machine learning algorithms for data analysis and quantification, has made it feasible to conduct large cohort studies for novel early diseases biomarker discovery.

In this study, various workflows for plasma proteomic profiling were compared to establish a methodology characterized by sensitivity, reproducibility, and depth. Our results demonstrated that the SeerProteographXT outperformed other methods, identifying and quantifying over 4.2-fold more protein groups compared to NeatPlasma and 2.4-fold more compared to PreOmicsEnrich-iST. Similarly, peptides were identified at a higher rate, with SeerProteographXT quantifying over 6.7-fold more compared to NeatPlasma and 4-fold more compared to PreOmicsEnrich-iST. The SeerProteographXT workflow’s full automation improved the median coefficients of variation (CV) to 10.7%, compared to 24.6% for NeatPlasma and 21.0% for PreOmicsEnrich-ST. The enhanced depth and dynamic range reduction achieved by the SeerProteographXT workflow are crucial for detecting extremely low-abundance proteins. Functional analysis using Gene Ontology (GO) terms and pathway analysis revealed associations with receptors, cytokines, interleukins, immune responses, TNF alpha signaling pathway, and T and B cell receptor signaling, suggesting the potential of these proteins as critical biomarkers.

In conclusion, the SeerProteographXT workflow, characterized by its sensitivity, reproducibility, and ability to provide deep proteome coverage with state-of-the-art mass spectrometers, could significantly contribute to the discovery of novel early disease biomarkers through large cohort studies across various diseases such as cancer, neurological disorders, and cardiovascular conditions.

## Data Availability

The raw data (diaPASEF) files have been uploaded to Pride and are accessible in Pride PXD050425.
